# Anticonvulsants for Psychiatric Disorders in Children and Adolescents: A Systematic Review of Their Efficacy

**DOI:** 10.3389/fpsyt.2018.00270

**Published:** 2018-06-22

**Authors:** Chiara Davico, Carlotta Canavese, Roberta Vittorini, Marina Gandione, Benedetto Vitiello

**Affiliations:** Division of Child and Adolescent Neuropsychiatry, Department of Public Health and Pediatric Sciences, University of Turin, Turin, Italy

**Keywords:** anticonvulsants, children, psychiatric, bipolar, aggression, clinical trial

## Abstract

**Aim:** Anticonvulsant medications are frequently used in clinical practice to treat psychiatric disorders in children and adolescents, but the evidence for their efficacy is uncertain. We conducted a systematic review of published randomized controlled trials (RCT) that assessed the psychiatric benefit of anticonvulsants in patients under 18 years of age.

**Method:** The Medline, Scopus, Web of Science, and ClinicalTrials.gov databases were systematically searched for peer-reviewed primary publications of RCTs with a minimum of 10 patients per treatment arm through December 2017.

**Results:** Out of 355 identified non-duplicative publications, 24 met the inclusion criteria. Most RCTs were to treat bipolar disorder (*n* = 12) or manage recurrent aggression (*n* = 9). Few (*n* = 3) had both a multisite design and adequate statistical power. Valproate was the most frequently studied anticonvulsant (*n* = 15). Out of three placebo-controlled RCTs of valproate in bipolar disorder, none showed efficacy. In four RCTs, valproate was inferior to the antipsychotic risperidone. In several small, single-site RCTs, valproate and sulthiame were better than placebo for the management of recurrent aggression.

**Conclusions:** Currently available RCTs do not support the efficacy of anticonvulsants as mood stabilizers in children. There is some preliminary evidence from small RCTs of the efficacy of some anticonvulsants in the control of aggression and behavioral dyscontrol in conduct disorder, autism, and intellectual disability.

## Introduction

Anticonvulsant medications have been used for decades in the treatment of psychiatric disorders. It is postulated that the biochemical mechanisms underlying their anti-seizure activity can lead also to stabilization of mood and behavior (1). In adults, valproate, carbamazepine, and lamotrigine have demonstrated efficacy as mood stabilizers in acute mania and/or as maintenance treatment of bipolar disorder to prevent recurrence (2–5). Oxcarbazepine and topiramate are also used, but without clear-cut evidence of efficacy (6–8). In addition, some anticonvulsants have anti-aggressive properties, and carbamazepine, oxcarbazepine, and phenytoin have been found to be effective in the management of recurrent impulsive aggression (9).

In children (here intended as individuals under 18 years of age), anticonvulsants are frequently used to stabilize mood and behavior, usually in the context of bipolar disorder or other disorders that are accompanied by recurrent aggression, self-injury, or severe temper dysregulation, such as intellectual disability, autism spectrum disorder, conduct disorder, and attention deficit-hyperactivity disorder (ADHD) (10). In fact, anticonvulsants have been among the most commonly used pharmacological agents in pediatric bipolar disorder (11).

No anticonvulsant currently carries regulatory approval for pediatric use for the treatment of bipolar disorder or other psychiatric indications. Thus, anticonvulsants are used “off label” in children. Uncontrolled investigations have been indeed suggestive of efficacy (12). Uncontrolled studies, however, cannot constitute evidence of efficacy, especially in psychiatric conditions, such as mood disorders, that are characterized by high rates of spontaneous improvement and placebo effect. Only randomized controlled trials (RCTs) can demonstrate efficacy.

In order to evaluate the evidence for the efficacy of anticonvulsants in the treatment of psychiatric disorders in children, we conducted a systematic review of RCTs. The main aim was to identify which anticonvulsants, if any, have proven efficacy in the treatment of psychiatric disorders in children. According to evidence-based medicine standards, efficacy would be proven if supported by at least two independent RCTs.

## Methods

The standard methodology of systematic reviews was applied (13).

### Selection criteria

We searched for English language, peer-reviewed publications that were the primary reports of RCTs testing the efficacy of anticonvulsants in the treatment of psychiatric disorders in children. Included were all anticonvulsants with proven anticonvulsant effects and currently approved for the treatment of epilepsy. The psychiatric conditions included: mood disorders (depression and bipolar disorder), conduct disorder, recurrent aggression, ADHD, anxiety, autism spectrum disorder, eating disorders, and tic disorders. Excluded was the use of anticonvulsants for migraine, headache, neuropathy, or pain management. Excluded were also RCTs in which the anticonvulsant was not the independent variable being tested for efficacy. A minimum sample size of 10 children randomized to each treatment group was required for inclusion. RCTs that enrolled adults, in addition to children, were included only if the study sample had a preponderance of subjects under 18 years of age.

### Search mechanism

The Medline, Web of Science, and Scopus databases were systematically searched for English language publications through December 2017. The search inputs were: “anticonvulsant and children (age 0–17 years) and psychiatric disorder or bipolar disorder or mania or depression or anxiety or aggression or autism or conduct disorder or ADHD or Tourette or eating disorder,” repeated for specific anticonvulsant medication (i.e., valproate, carbamazepine, oxcarbazepine, lamotrigine, phenytoin, topiramate, gabapentin, pregabalin, levetiracetam, clonazepam, clobazam, perampanel). All searches used *clinical trial* as a filter. In addition, the ClinicalTrial.gov database was similarly searched for clinical trials of anticonvulsants in children for bipolar disorder, anxiety, ADHD, and autism.

### Review and selection process

After removal of duplicates, the publication titles and abstracts were visually inspected and reviewed independently based on the selection criteria by two experts (CD and BV). Disagreements were discussed and resolved by consensus in order to arrive at an agreed upon list of RCT publications.

### Analysis

A qualitative analysis of the selected studies was independently conducted, based on the study reported characteristics and outcomes. The characteristics, quality, and limitations of each selected RCT were assessed based on the published report. Disagreements between raters were resolved by discussion and consensus. When additional information was needed, the corresponding author of the report was contacted in an attempt to acquire additional information. In assessing quality of each study, the presence of the following seven elements was examined: (1) double-blind design; (2) description of randomization and masking methods to minimize assessment biases; (3) multiple clinical sites (more than one); (4) sample size of at least 40 children randomized to each treatment group to provide statistical power to detect medium treatment effect sizes; (5) sufficient exposure to treatment with respect to dosage (i.e., dose in the known therapeutic range for anticonvulsant action, supported by plasma levels when available) and duration of treatment (at least 3 weeks for acute efficacy and at least 12 weeks for efficacy of maintenance treatment); (6) acceptable sample retention (< 25% of the randomized sample lost to follow-up); and (7) intent-to-treat analyses.

## Results

**Figure 1:**
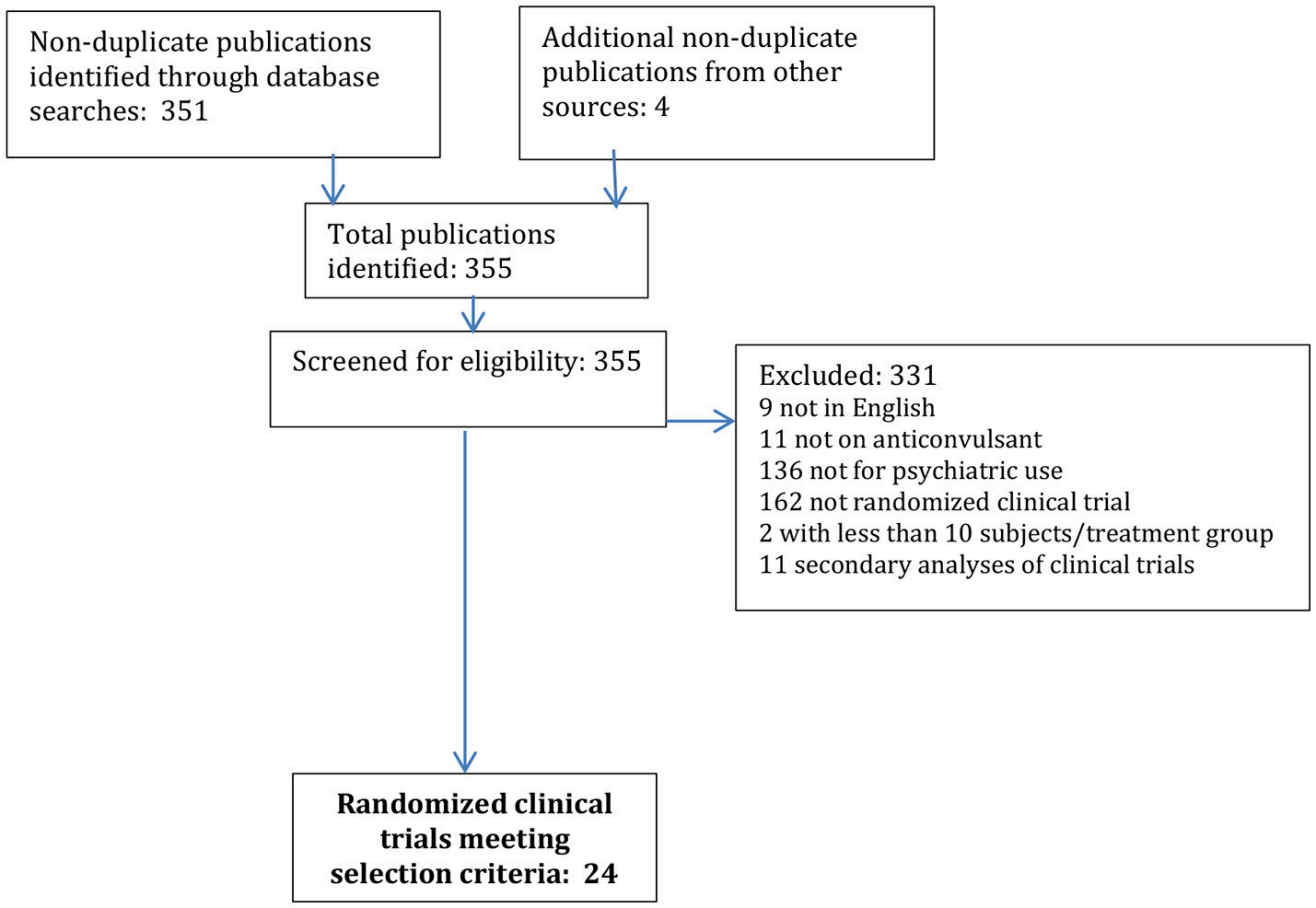
Review flowchart.

The initial search yielded a total of 351 non-duplicate publications; an additional 4 publications were identified through manual reference review or other sources. Of these 355 publications, 331 were excluded as not meeting the pre-specified selection criteria, being 9 not in English, 11 not on anticonvulsants, 136 not for psychiatric use, 162 not RCT, 2 with too small of a sample size, and 11 secondary analyses of RCT (see Figure 1).

A total of 24 publications, each constituting the primary report of a RCT of anticonvulsant efficacy in psychiatric disorder in children were identified (see Tables 1, 2). Half of these RCTs were in the treatment of bipolar disorder, including acute control of manic/mixed episodes and chronic maintenance to prevent recurrence (see Table 3). The other most common psychiatric use was for the control of recurrent impulsive aggression, mainly in the context of a neurodevelopmental disorder, such as autism and/or intellectual disability (*n* = 9).

**Table 1:** Randomized controlled clinical trials assessing the efficacy of anticonvulsant medications in the treatment of children (under 18 years of age) with bipolar disorder (*n* = 12)^a^.

**Medications**	**Psychiatric disorder**	**Target**	**Design**	**Sample**	**Outcome measures**	**Dosage/Serum level**	**Results^b^**	**References**
Valproate	Bipolar I or II disorder	Maintenance of mood stability	Discontinuation design: after stabilization on Li plus DVPX, randomization to Li or DVPX monotherapy for 18 months	*n* = 60 age:5–17 y	Time to relapse (mood instability)	DVPX: 20 mg/kg/d. Serum valproic acid level: 75 mcg/mL (mean)	No difference between Li and DVPX	Findling et al. 14
Valproate	Bipolar I acute mania or mixed episode	Mania, irritability	Randomization to DVPX or quetiapine for 4 weeks	*n* = 50 age:12–18 y	YMRS	Serum valproic acid level: 80–120 mcg/mL	No difference between DVPX and quetiapine on YMRS scores. More rapid symptom decrease with quetiapine	DelBello et al. 15
Valproate	Bipolar NOS, cyclothymia	Maintenance of mood stability	Randomization to DVPX or placebo for up to 5 years	*n* = 56 age:5–17 y	Time to drug discontinuation	Up to 15 mg/kg/d (maximum: 1250 mg/d)	No difference between DVPX and placebo	Findling et al. 16
Valproate	Bipolar I acute mania or mixed episode	Mania, irritability	Randomization to DVPX or placebo for 4 weeks	*n* = 150 age:10–17 y	YMRS CGI-I CGI-S	DVPX: 1,286 mg (mean). Serum valproic acid level: 80 mcg/mL (mean)	No difference between DVPX and placebo.	Wagner et al. 17
Valproate	Bipolar I acute mania or mixed episode	Mania, irritability	Randomization to DVPX or risperidone for 6 weeks	*n* = 66 age: 8–18 y	YMRS	DVPX: up to serum valproic acid level of 60–120 mcg/mL	Risperidone superior to DVPX	Pavuluri et al. 18
Valproate	Bipolar I acute mania or mixed episode	Mania, irritability	Randomization to DVPX, lithium or risperidone for 8 weeks. Open study, with blinded raters	*n* = 290 age: 6–15 y	CGI-I for bipolar symptoms	Serum valproic acid level: 113.6 mcg/mL (mean)	Risperidone superior to DVPX and lithium. No difference between DVPX and lithium	Geller et al. 19
Valproate	Bipolar I acute mania or mixed episode	Mania, irritability	Randomization to switching to or adding DVPX, lithium or risperidone for 8 weeks. Open study, with blinded raters	*n* = 154 age: 6–15 y	CGI-I for bipolar symptoms	Up to valproic acid serum levels of 111–125 mcg/mL	Risperidone superior to DVPX and lithium	Walkup et al. 20
Valproate	Bipolar I acute mania or mixed episode	Mania, irritability	Randomization to valproate, risperidone, or placebo for 6 weeks	*n* = 46 age: 3–7 y	YMRS	Up to valproic acid serum levels of 80–100 mcg/mL	No difference between valproate and placebo. Risperidone superior to placebo.	Kowatch et al. 21
Valproate, Topiramate	Bipolar I acute mania	Mania	Randomization to DVPX or topiramate for 8 weeks	*n* = 142 age: 12–18 y	YMRS	Valproate: up to 1,200 mg/d	Valproate superior to topiramate	Hebrani et al. 22
Valproate, Carbamazepine	Bipolar I or II disorder, mixed or manic episode	Mania, irritability	Randomization to DVPX, carbamazepine or, lithium. Open study with blinded raters	*n* = 42 age: 11 y (mean)	YMRS	Up to serum valproic acid level of 85–110 mcg/mL and carbamazepine level of 7–10 mcg/mL	No difference between treatment groups	Kowatch et al. 23
Oxcarbazepine	Bipolar I acute mania or mixed episode	Mania, irritability	Randomization to oxcarbazepine or placebo for 7 weeks	*n* = 116 age:7–18 y	YMRS	900–2,400 mg/d (mean 1,515)	No difference between oxcarbazepine and placebo	Wagner et al. 24
Lamotrigine	Bipolar I disorder	Maintenance of mood stability	Discontination study: after initial stabilization with other mood stabilizer plus lamotrigine, randomization to continuing lamotrigine or switching to placebo, for 36 weeks	*n* = 60 age:10–17 y	Time to occurrence of a bipolar event	Up to 240 mg/d	No difference between lamotrigine and placebo (secondary analysis: lamotrigine superior to placebo for 13–17 year old subgroup)	Findling et al. (25)

a Double-blind masking unless otherwise specified.

b Statistically significant differences at p ≤ 0.05.

*Bipolar NOS, bipolar disorder not otherwise specified (Diagnostic and Statistical Manual - IV edition); CGI-I, Clinical Global Impression-Improvement Scale; CGI-S, Clinical Global Impression-Severity Scale; d, day; DVPX, divalproex (formulation of valproate); Li, lithium; y, year; YMRS, Young Mania Rating Scale*.

**Table 2:** Randomized controlled clinical trials assessing the efficacy of anticonvulsant medications in the treatment of children (under 18 years of age) with psychiatric disorders other than bipolar disorder (*n* = 12)^a^.

**Medications**	**Psychiatric disorder**	**Target**	**Design**	**Sample**	**Outcome measures**	**Dosage/serum level**	**Results^b^**	**References**
Valproate	ODD or CD	Explosive temper, mood lability, aggression	Randomization to DVPX or placebo for 6 weeks (phase 1) followed by cross-over to other treatment for 6 weeks (phase 2)	*n* = 20 age: 10–18 y	Modified Overt Aggression Scale	DVPX: 750-1,500 mg/d	DVPX superior to placebo in phase 1. No difference in phase 2	Donovan et al. 26
Valproate	CD	Explosive temper, mood lability, aggression	Randomization to low or high dose of valproate for 7 weeks	*n* = 71 age: 16 y (mean)	CGI-S CGI-I	Low dose: up to 250 mg/d High dose: 500-1,500 mg/d	High dose of valproate superior to lower dose	Steiner et al. 27
Valproate	ADHD with ODD or CD	Aggression	Initial treatment with stimulant monotherapy, followed by randomization to DVPX or placebo for 8 weeks	*n* = 30 age: 6–13 y	Retrospective-Modified Overt Aggression Scale	20 mg/kg/d. Valproic acid serum level: 68.1 mcg/mL (mean)	DVPX superior to placebo	Blader et al. 28
Valproate	Autism spectrum disorder	Aggression	Randomization to valproate or placebo for 8 weeks	*n* = 30 age: 6–20 y	ABC CGI-I	Valproic acid serum level: 77.8 mcg/mL (mean)	No difference between valproate and placebo	Hellings et al. 29
Valproate	Autism spectrum disorder	Irritability/ Aggression	Randomization to DVPX or placebo for 12 weeks	*n* = 27 age: 5–17 y	ABC CGI-I	DVPX: up to 1,000 mg/d	DVPX superior to placebo	Hollander et al. 30
Carbamazepine	CD	Aggression	Randomization to carbamazepine or placebo for 6 weeks	*n* = 22 age: 5–12 y	Overt Aggression Scale, CGI, Children's Psychiatric Rating Scale	200–800 mg/d (mean 683) Serum carbamazepine levels: 5.0-9.1 mcg/mL	No difference between carbamazepine and placebo	Cueva et al. 31
Carbamazepine	ADHD	ADHD symptoms	Randomization to carbamazepine or clonidine for 4 weeks	*n* = 50 age: 4–12 y	Vanderbilt ADHD Rating Scale	Unspecified	Clonidine superior to carbamazepine	Nair and Mahadevan, 32
Levetiracetam	Tourette disorder	Tics	Within-subject, crossover with randomization to levetiracetam or placebo, sequentially, for 4 weeks each	*n* = 22 age: 8–16 y	Yale Global Tic Severity Scale	Up to 30 mg/kg/d	No difference between levetiracetam and placebo	Smith-Hicks et al. 33
Levetiracetam	Autism spectrum disorder	Hyperactivity, impulsivity,aggression, and mood lability	Randomization to levetiracetam or placebo for 10 weeks	*n* = 20 age:5–17 y	CGI-I, ABC, Conners' Rating Scale-Revised	863 mg/d (mean)	No difference between levetiracetam and placebo	Wasserman et al. 34
Clonazepam	Anxiety disorders	Decrease in anxiety symptoms	Within-subject, crossover with randomization to clonazepam or placebo, sequentially, each for 4 weeks	*n* = 15 age: 7–13 y	Children Manifest Anxiety Scale	Up to 2 mg/d	No difference between clonazepam and placebo	Graae et al. 35
Sulthiame	Intellectual disability	Hyperactivity, aggression	Within-subject, crossover with randomization to sulthiame or placebo, sequentially, each for 6 weeks	*n* = 42 age:7–38 y (mean 17)	Behavior rating scale	Up to 600 mg/d	Sulthiame superior to placebo	Moffat et al. 36
Sulthiame	Intellectual disability	Hyperactivity, aggression	Randomization to sulthiame or placebo for 14 weeks	*n* = 34 age: 6–24 y	Behavior rating scale	Up to 15 mg/kg/d	Sulthiame superior to placebo	Al-Kaisi and McGuire, 37

a Double-blind masking unless otherwise specified.

b Statistically significant differences at p ≤ 0.05.

*ABC, Aberrant Behavior Checklist; ADHD, attention deficit/hyperactivity disorder; CD, conduct disorder; ODD, oppositional defiant disorder*.

**Table 3:** Primary target of the 24 randomized controlled clinical trials (RCTs) of anticonvulsant medications in psychiatric disorders in children (under 18 years of age).

	**no of RCTs**
Control of acute symptoms of mania and irritability in bipolar disorder	9
Prevention of recurrent explosive aggression	9
Prevention of recurrence of bipolar acute episode	3
Control of symptoms of ADHD	1
Control of tics in tourette disorder	1
Control of symptoms of anxiety	1

The RCTs were conducted in double-blind conditions, except for four, which, however, employed masking methods (i.e., blinded raters) to limit ascertainment biases. Most were placebo-controlled, while four were comparative effectiveness RCTs of different active medications without a placebo control. The age of the RCT samples was mainly between 5 and 17 years. Only two RCTs included preschoolers as young as 3 years of age (21, 32).

Treatment exposure, with respect to adequate dosage and sufficient duration, as well as retention and statistical analyses, were considered to be satisfactory, but only five RCT involved more than one site and only five had a sample size of at least 40 subjects per treatment group (see Supplementary Table 1). Of the 24 RCTs, 13 (54%) did not find a statistically significant difference (Tables 1, 2).

Only two RCTs were deemed to have met all the specified seven quality elements (17, 24), and, in particular, to have adequate sample size. Neither of these studies found the anticonvulsant medication to be better than placebo.

Most of the RCTs evaluated valproate (*n* = 15), while three tested carbamazepine (one of these studies included also valproate). The remaining anticonvulsants (oxcarbazepine, lamotrigine, levetiracetam, clonazepam, topiramate, and sulthiame) had only one or two RCTs each. Valproate was tested as a mood stabilizer in bipolar disorder in 10 RCTs and in the prevention of recurrent aggression in five RCTs. In bipolar disorder, none of the three placebo-controlled RCTs showed efficacy (16, 17, 21). Four RCTs showed superiority of the antipsychotic risperidone over valproate (18–21). No difference was detected between valproate and lithium (14), carbamazepine (23), or quetiapine (15). Finally, one RCT conducted to test the antimanic effects of topiramate in hospitalized youths, using valproate as a comparison group, found valproate to be superior to topiramate (22).

As anti-aggressive agent, valproate showed no difference from placebo in one RCT (29) and was better than placebo in three small RCTs, one in children with autism spectrum disorder (30) and two in children with conduct disorder or ADHD (26, 28). One RCT that compared high with low dose of valproate found superiority of the higher dose (27).

Four RCTs tested carbamazepine or oxcarbazepine in the treatment of mania, aggression, or ADHD. Two of these RCTs found no difference from placebo (24, 31). Another RCT found carbamazepine inferior to clonidine in ADHD (32), and in the third one there was no difference vs. valproate (23).

No evidence of efficacy emerged for lamotrigine, levetiracetam, and clonazepam. In two RCTs that were conducted more than 40 years ago in institutionalized, severely impaired subjects with intellectual disability, including both youths and adults, sulthiame, was better than placebo for controlling aggression and hyperactivity (36, 37).

### Conclusions

This systematic review identified mostly small controlled studies with important methodological limitations and heterogeneity with respect to type of medication and clinical target. No evidence emerged for the efficacy of anticonvulsants in children with bipolar disorder. There is limited evidence for the efficacy of valproate and sulthiame for the management of aggressive behavior. For sulthiame studies were conducted in samples that included adult patients and the specific efficacy in children cannot be estimated.

## Discussion

To evaluate the evidence for efficacy of anticonvulsant medications in psychiatric disorders of childhood, we conducted a systematic qualitative review of relevant published RCTs. We restricted the search to RCTs because uncontrolled studies cannot provide evidence of treatment effects given the variable placebo-response in psychiatric conditions.

Twenty-four RCTs met the pre-specified selection criteria (Tables 1, 2). The medication dosage and the duration of treatment were generally appropriate, and many studies measured medication serum levels. Most of these RCTs, however, had important methodological limitations, especially a small sample size (< 40 per treatment group), and therefore inadequate statistical power to detect medium effect sizes (see Supplementary Table 1).

A sample size of 40 subjects per treatment group will provide 80% statistical power to detect a between-group effect size usually considered in the medium range (e.g., a Cohen's *d* = 0.6) as statistically significant at a *p* ≤ 0.05 (38). The small sample size of the large majority of these RCTs strongly limits their capacity to identify statistically significant treatment differences. In fact, out of 24 RCTs, 13 (54%) did not find a statistically significant difference between the treatment groups.

A considerable number of RCTs was conducted on valproate (*n* = 15), three of which had adequate sample size, while few studies were devoted to other anticonvulsants. In the treatment of children with bipolar disorder, valproate showed no evidence of superiority over placebo, and was actually inferior to risperidone based on four RCT (Table 1).

Two RCTs compared valproate to lithium or quetiapine, respectively, in bipolar disorder, and found no difference between treatment groups (14, 15). Considering the small sample size of these RCTs and the lack of a placebo condition, the lack of difference cannot be interpreted as evidence of efficacy. Another, single-site, RCT compared topiramate to valproate in hospitalized, acutely manic youths (22). This study, which had been designed to test the efficacy of topiramate, found valproate to be superior to topiramate. The report, however, lacks an adequate description of the masking methods, and the especially large effect size is surprising and possibly due to the specific context of the hospital where the study was conducted.

These data on anticonvulsants in child bipolar disorder appear to be at odds with the evidence for efficacy of valproate, carbamazepine, and lamotrigine in adults with bipolar disorder. In adults, valproate and carbamazepine are superior to placebo in acute mania (2, 3), although less effective than antipsychotics (3), and lamotrigine is superior to placebo in bipolar depression (4). More limited evidence supports also the efficacy of valproate as maintenance treatment in adult bipolar disorder (5). The discrepancy between adults and children is suggestive of developmental differences in the psychopathology of the mood dysregulation. It should also be pointed out that there are several psychiatric medications, such as antidepressants and benzodiazepines, whose efficacy has been shown in adults but not in children (39).

For the management of aggression and explosive temper, valproate showed efficacy in four small and single-site RCTs in children with conduct disorder, ADHD, or autism (26–28, 30). One of these, used valproate as add-on treatment to stimulant medication in ADHD (28). Another one was designed as a crossover trial, but could not complete the second segment of the study and analyzed only the first part (26). Even if none of these studies met all the methodological quality criteria, these data can be taken as tentative evidence of efficacy of valproate in controlling aggressive behavior. This is consistent with the results of a meta-analysis of anticonvulsants in the management of aggression in adults (9). These findings, however, should be confirmed by adequately powered, multisite RCTs.

Little evidence of efficacy in bipolar disorder emerged from the RCTs of other commonly used anticonvulsants. In particular, a statistically powered, multi-site, placebo-controlled trial of oxcarbazepine in bipolar disorder found no statistically significant difference (24). Lamotrigine, which is effective in bipolar depression in adults, did no better than placebo in a RCT that included children aged from 10 to 17 years, although secondary analyses found superiority in the 13- to 17-year-old subgroup (26). Sulthiame, an infrequently used anticonvulsant, was found better than placebo in 2 RCTs conducted more than 40 years ago for the control of aggression and hyperactivity in institutionalized patients, including both youths and adults, with severe intellectual disability (36, 37), but the implications of these data for current practice are unclear.

The limitations of this review are primarily related to the design characteristics of the studies and especially to the small sample size of most of them. The studies are also rather heterogeneous with respect to both the type of anticonvulsant being tested and the clinical indication being targeted. Another limitation is the relatively wide range of patient age in most studies, some of which included also adults.

Based on the current data, the psychiatric use of anticonvulsants in children cannot be supported according to evidence-based standards. Their potential benefit valproate and sulthiame for the management of recurrent aggression, however, cannot be discounted. The potential benefit of these medications must be in any case balanced against the risk for adverse effects, including psychiatric ones (40).

In conclusion, the efficacy of anticonvulsants as mood stabilizers in children with bipolar disorder remains unproven. There is limited evidence that some anticonvulsants may decrease aggressive behavior and explosive temper, especially in patients with neurodevelopmental disorders and intellectual disability. Because this evidence comes mainly from small studies, it might be informative to conduct more precisely designed and adequately powered RCT targeting recurrent and impulsive aggression in children with neurodevelopmental disorders.

## Author contributions

BV and CD were responsible for review, data extraction, and evaluation. CC, RV, and MG contributed expert review and helped in the data interpretation and manuscript preparation.

### Conflict of interest statement

The authors declare that the research was conducted in the absence of any commercial or financial relationships that could be construed as a potential conflict of interest.
