# Supplementary material for: Anticonvulsants for Psychiatric Disorders in Children and Adolescents: A Systematic Review of Their Efficacy
Source: Front Psychiatry. 2018 Jun 22;9:270. doi: 10.3389/fpsyt.2018.00270 (PMC6024111; doi:10.3389/fpsyt.2018.00270)
Supplement: Supplementary file 1 [file Table_1.docx]

Supplemental Table 1

**Strengths and limitations of randomized controlled clinical trials assessing the efficacy of anticonvulsant medications in the treatment of psychiatric disorders in children (under 18 years of age)**

| Medication | Psychiatric disorder | Study Reference | Double-blind | Description of randomization and masking methods | Multisite | Sample size n>40 per group | Sufficient exposure to treatment^a^ | Acceptable retention | Intent-to-treat analyses | Total quality criteria met out of possible 7 |
| --- | --- | --- | --- | --- | --- | --- | --- | --- | --- | --- |
| **Valproate** | Bipolar I or II disorder | Findling et al. 2005 (14) | yes | yes | no | no | yes | yes | yes | 5 |
| **Valproate** | Bipolar I acute mania or mixed episode | DelBello et al. 2006  (15) | yes | yes | no | no | yes | yes | yes | 5 |
| **Valproate** | Bipolar NOS | Findling et al. 2007  (16) | yes | yes | no | no | yes | yes | yes | 5 |
| **Valproate** | Bipolar I acute mania or mixed episode | Wagner et al. 2009  (17) | yes | yes | yes | yes | yes | yes | yes | 7 |
| **Valproate** | Bipolar I acute mania or mixed episode | Pavuluri et al. 2010  (18) | yes | yes | no | no | yes | yes | yes | 5 |
| **Valproate** | Bipolar I acute mania or mixed episode | Geller et al. 2012  (19) | no | yes | yes | yes | yes | yes | yes | 6 |
| **Valproate** | Bipolar I acute mania or mixed episode | Walkup et al. 2015  (20) | no | yes | yes | no | yes | yes | yes | 5 |
| **Valproate** | Bipolar I acute mania or mixed episode | Kowatch et al. 2015  (21) | yes | yes | yes | no | yes. | yes | yes | 6 |
| **Valproate, Topiramate** | Bipolar I acute mania | Hebrani et al. 2009  (22) | yes | no | no | yes | yes | yes | yes | 5 |
| **Valproate,**  **Carbamazepine** | Bipolar I or II disorder, mixed or manic episode | Kowatch et al. 2000  (23) | no | yes | no | no | yes | yes | yes | 4 |
| **Oxcarbazepine** | Bipolar I acute mania or mixed episode | Wagner et al. 2006  (24) | yes | yes | yes | yes | yes | yes | yes | 7 |
| **Lamotrigine** | Bipolar I disorder | Findling et al. 2015  (25) | yes | yes | no | no | yes | yes | yes | 5 |
| **Valproate** | ODD or CD | Donovan et al. 2000  (26) | yes | yes | no | no | yes | yes | no | 4 |
| **Valproate** | CD | Steiner et al. 2003  (27) | no | yes | no | no | yes | yes | yes | 4 |
| **Valproate** | ADHD with ODD or CD | Blader et al. 2009  (28) | yes | yes | no | no | yes | yes | yes | 5 |
| **Valproate** | Autism spectrum disorder | Hellings et al. 2005  (29) | yes | yes | no | no | yes | yes | yes | 5 |
| **Valproate** | Autism spectrum disorder | Hollander et al. 2010  (30) | yes | yes | no | no | yes | yes | yes | 5 |
| **Carbamazepine** | CD | Cueva et al. 1996  (31) | yes | yes | no | no | yes | yes | yes | 5 |
| **Carbamazepine** | ADHD | Nair & Mahadevet al. 2009 (32) | yes | no | no | no | yes | yes | yes | 4 |
| **Levetiracetam** | Tourette  Disorder | Smith-Hicks et al. 2007  (33) | yes | yes | no | no | yes | yes | yes | 5 |
| **Levetiracetam** | Autism spectrum disorder | Wasserman et al. 2006  (34) | yes | yes | no | no | yes | yes | yes | 5 |
| **Clonazepam** | Anxiety disorders | Graae et al. 1994  (35) | yes | yes | no | no | yes | yes | yes | 5 |
| **Sulthiame** | Intellectual disability | Moffat et al. 1970  (36) | yes | yes | no | yes | yes | yes | yes | 6 |
| **Sulthiame** | Intellectual disability | Al-Kaisi & McGuire, 1974  (37) | yes | yes | no | no | yes | yes | yes | 5 |

^a^Based on adequate dose (i.e., dose in the known therapeutic range for anticonvulsant action, supported by plasma levels when available) and duration of treatment (at least 3 weeks for acute efficacy and at least 12 weeks for maintenance treatment).
